# Acute Kidney Injury and Gut Dysbiosis: A Narrative Review Focus on Pathophysiology and Treatment

**DOI:** 10.3390/ijms23073658

**Published:** 2022-03-26

**Authors:** Yu-Ting Chou, Wei-Chih Kan, Chih-Chung Shiao

**Affiliations:** 1 Department of Internal Medicine, National Taiwan University Hospital, Taipei 100225, Taiwan; elliechou0821@gmail.com; 2 Department of Nephrology, Department of Internal Medicine, Chi Mei Medical Center, Tainan 71004, Taiwan; 3 Department of Biological Science and Technology, Chung Hwa University of Medical Technology, Tainan 71703, Taiwan; 4 Division of Nephrology, Department of Internal Medicine, Camillian Saint Mary’s Hospital Luodong, Yilan 265, Taiwan; 5 Saint Mary’s Junior College of Medicine, Nursing and Management, Yilan 26647, Taiwan

**Keywords:** acute kidney injury, dysbiosis, immunity, prebiotics, probiotics, short-chain fatty acids, synbiotics

## Abstract

Acute kidney injury (AKI) and gut dysbiosis affect each other bidirectionally. AKI induces microbiota alteration in the gastrointestinal (GI) system, while gut dysbiosis also aggravates AKI. The interplay between AKI and gut dysbiosis is not yet well clarified but worthy of further investigation. The current review focuses on the pathophysiology of this bidirectional interplay and AKI treatment in this base. Both macrophages and neutrophils of the innate immunity and the T helper type 17 cell from the adaptive immunity are the critical players of AKI-induced gut dysbiosis. Conversely, dysbiosis-induced overproduction of gut-derived uremic toxins and insufficient generation of short-chain fatty acids are the main factors deteriorating AKI. Many novel treatments are proposed to deter AKI progression by reforming the GI microbiome and breaking this vicious cycle. Data support the benefits of probiotic treatment in AKI patients, while the results of postbiotics are mainly limited to animals. Prebiotics and synbiotics are primarily discussed in chronic kidney disease patients rather than AKI patients. The effect of adsorbent treatment seems promising, but more studies are required before the treatment can be applied to patients. Immune therapy and some repurposed drugs such as allopurinol are prospects of future treatments and are worth more discussion and survey.

## 1. Introduction

Acute kidney injury (AKI) is a common clinical entity affecting up to 1% of the general population, 2–7% of hospitalizations, and 13–78% of critically ill patients [1,2,3,4]. It is associated with widely ranged morbidity and mortality risks that increase concurrently with increasing severity and duration of AKI [5,6]. Despite improving medicine, AKI-associated morbidity and mortality remain high [7]. One probable explanation for the discouraging results is the organ crosstalk between kidney and extra-renal organs during AKI [8]. The existing studies have demonstrated the crosstalks between the kidney and many remote organs/systems such as the heart, brain, immune system, lung, gut, bone. These organ crosstalks result in many long-term distant organ consequences following AKI, including coronary event, upper gastrointestinal (GI) bleeding, incident stroke, malignancy, and even bone fracture [9].

The GI tract and the surrounding mesenteric lymphatics comprise the body’s largest absorptive surface. It exposes nutrients and foreign antigens, microbes, and potentially harmful elements. Multilayered mechanisms from mechanical epithelial tight junction to gut flora and immune cells equip our body with a defensive barrier of intrusion [10]. Early in the 2000s, studies drew the link between the GI tract and multiple organ dysfunction syndromes (MODS) and proposed that the gut acts as a motor of organ dysfunction [11]. They also pointed out that disruption of the intestinal epithelium barrier leads to bacteria and microbial products leaking, initiating, and exacerbating the development of MODS.

In recent years, evidence has started to show that the kidney and GI tract have crossed paths. The interplay between gut microbiota and the kidney has provoked new research and much discussion in pathophysiology and treatments. However, the studies evaluating the association between gut microbiota alteration, so-called dysbiosis, and kidney disease are mainly tackled in chronic kidney disease (CKD) [12,13], whereas limited research is available regarding the association between AKI and gut dysbiosis (Table 1) [14,15,16,17]. These experimental investigations found that gut microbiota plays nephroprotective or neuropathogenic roles in AKI [15,16]. Clinically, AKI induces gut dysbiosis that includes the disturbance of *Escherichia coli*, *Bacteroidetes*, *Bifidobacterium, Salmonella*, *Lactobacillus*, *Clostridium*, *Ruminococcus*, *Rothia*, *Staphylococcus*, *Enterobacter*, *Faecalibacterium*, and *Lachnospiraceae*, etc. [14,15,16,17]. The AKI-induced gut dysbiosis alters the metabolism of many short-chain fatty acids (SCFAs), amino acids such as D-serine, and some biological intermediates such as acylcarnitines [15,16,17]. Gut dysbiosis also results in intestinal inflammation and leaky gut. All the factors mentioned above play crucial roles in determining AKI development and severity. Briefly speaking, the bidirectional interplay between kidney and gut dysbiosis means that one affects and deteriorates the other, explaining the complexity and rapid downhill of the process.

However, the pathophysiological mechanism between AKI and gut dysbiosis is not clearly understood and is worthy of a comprehensive and updated review. To improve knowledge and draw attention to the interplay of AKI and gut dysbiosis in the medical community, we provide a review article after an extensive search on PubMed using keywords of “gut” “dysbiosis” “kidney” “AKI” “pathophysiology” and “treatment”. The narrative review focuses on the pathophysiology of this interplay and potential AKI treatment. Although most proposed theories are based on animal models, this existing evidence helps physicians and researchers understand the underlying pathophysiology, which is crucial for new perspectives to improve prognoses of AKI patients.

## 2. Pathophysiology

**Figure 1:**
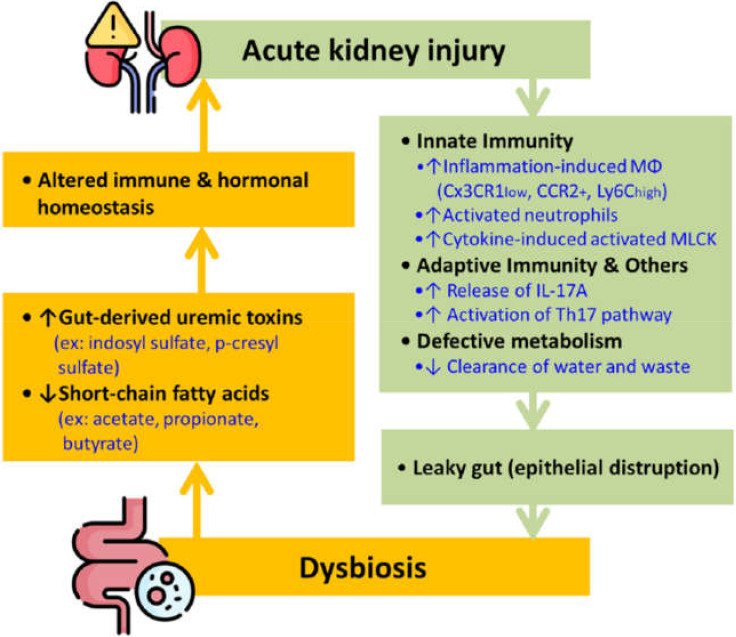
The bidirectional interplay between AKI and gut dysbiosis. **Note:** AKI prompts innate and adaptive immunity, disrupting the epithelial barrier and causing the gut microbiome imbalance. Dysbiosis can also cause breaking of immune and hormonal equilibrium that further worsens kidney function. **Abbreviation:** IL = interleukin; Mφ = macrophages, MLCK = myosin light chain kinase; Th17 = T helper type 17.

The term gut-kidney axis has drawn much attention in the past few years due to the fascinating interaction between the two organ systems. As shown in Figure 1, AKI sets off the immune responses, leading to epithelial disruption by activating macrophages and neutrophils of the innate immunity and the T helper type 17 (Th17) cell from the adaptive immunity. These processes result in a leaky gut that consequently causes dysbiosis. Studies demonstrated that AKI causes gut dysbiosis in 24 h [18]. Conversely, gut dysbiosis also causes an imbalance of different chemicals, specifically uremic toxins, and SCFAs, ultimately altering immune and hormonal homeostasis and causing a de novo or worsened AKI (Figure 1)

### 2.1. AKI Induces Gut Dysbiosis

Both innate immune and adaptive immune systems play pivotal roles in the process when AKI causes gut dysfunction. With the innate immune response, macrophages and neutrophils are the leading players. In an observation based on the murine model, macrophages that reside in normal kidney tissue stem from circulating Ly6C_high_ monocytes. They downregulate Ly6C expression, upregulate CX3CR1 receptors in a normal state, and produce CX3CR1_high_, CCR2^−^, and Ly6C_low_ macrophages [19]. The interaction between these Ly6C_low_ macrophages and endothelial cells tempers the expression of intracellular adhesion molecule 1, thereby preventing neutrophil infiltration and inflammation in ischemic renal injury [20]. Furthermore, Yang et al. exhibited increased Ly6C_low_ macrophages after IRI and identified these cells as M1-like proinflammatory macrophages by the rising nitric oxide synthase levels and declining arginase expression [17]. However, these molecules will express differently, and the macrophages will become Cx3CR1_low_, CCR2^+^, and Ly6C_high_ cells and lose their protective role under an inflammatory state [20]. The phenomenon also occurs in the situation of gut dysbiosis. The depletion of gut microbiota significantly compresses the expression of CX3CR1 and CCR2 in renal resident macrophages and bone marrow monocytes [21].

On the other hand, the neutrophil acts through Toll-Like Receptor (TLR) and Nod- Like Receptor (NLR) dependent pathways. The TLRs and NLRs recognize the antigens and activate the neutrophils when our bodies are exposed to pathogen-associated molecular patterns (PAMPs) and damage-associated molecular patterns (DAMPs). These activated neutrophils will secrete proteases, reactive oxygen species (ROS), and several lytic enzymes that directly break down the epithelial structure, mainly apical tight junctions and junctional adhesion molecules (JAM). The cytokines released from activated neutrophils also cause hyper-permeability by modifying the expression of JAM [22]. In addition, the cytokines-induced activated myosin light chain kinase (MLCK) phosphorylates the myosin light chain resulting in the contraction and further opening in the tight apical junction [23].

Although not clearly understood, several mechanisms are closely intertwined regarding the adaptive immune response between AKI and gut dysbiosis. The cytokine interleukin (IL)-17A is a distinctive route of the adaptive immune response. IL-17A mainly comes from Paneth cell degranulation and is essential in mediating kidney, intestine, and liver injury after AKI [24]. Another route is via the Th17 cells. The significant increase of IL17A1CD41 cells with unchanged interferon-gamma (IFN-γ)1CD41cell percentages suggests that the activation of the intestinal Th17 pathway is induced by kidney ischemia/reperfusion injury (IRI) [17]. A recent study also supports Th17’s role in the kidney inflammation process through the photoconversion of intestinal cells to track T cells. The Th17 cells, originally most abundant in the gut, migrate to the kidney in antineutrophil cytoplasmatic antibody (ANCA)-associated glomerulonephritis [25]. 

In addition to the immune responses, defective metabolism also directly causes harm to the normal gut structure. The inadequate clearance of water and metabolic waste (especially urea) from AKI is one culprit to intestinal dysbiosis [26]. Research has shown that injured kidneys with aggressive hydration treatment can worsen intestinal wall edema and destroy epithelial tight junction apparatus [27]. Furthermore, the retained urea due to the impaired renal function will be metabolized into ammonia and further converted into ammonium hydroxide, which disrupts tight junction proteins that bridge the epithelial gaps [27,28].

Speaking overall, AKI disturbs innate and adaptive immunities and metabolism by many pathways and subsequently damages the gut epithelial structure. Once this epithelial structure is disrupted, the gut becomes “leakier” and aggravates bacterial, inflammatory agents, and toxin translocation, causing dysbiosis.

### 2.2. Dysbiosis Exaggerates AKI

#### 2.2.1. Gut-Derived Uremic Toxins

Uremic toxins can cause harmful biochemical effects and accelerate renal failure by damaging tubular cells [29,30]. Several gut-derived uremic toxins, such as indoxyl sulfate (IS), p-cresyl sulfate (PCS), Trimethylamine-N-oxide (TMAO), indole-3 acetic acid, phenylacetylglutamine, etc., have been identified. IS and PCS are protein-bound, metabolic products of amino acids from anaerobic gut bacteria. IS is the product of dietary tryptophan metabolism, while PCS comes from phenylalanine and tyrosine [31,32,33]. TMAO is a small water-soluble molecule catabolized from quaternary amines like L-carnitine and phosphatidylcholine. TMAO is dialyzable because of its small, water-soluble qualities [34], but IS and PCS cannot be efficiently removed by conventional hemodialysis due to their high binding affinity to proteins [35]. This issue inspires the idea of an uremic toxin absorbent as a novel treatment for AKI.

IS and PCS are well-discussed toxins closely associated with cardiovascular events and mortality in hemodialysis patients [36,37]. The association between these two toxins and AKI has also been discovered recently. One prospective cohort study pointed out that serum IS levels are significantly elevated in patients with hospital-acquired AKI and are also linked to a worse prognosis [38]. Another clinical trial also found increased IS and PCS levels in AKI, which correlated with RIFLE classification [39]. As for TMAO, elevated TMAO levels are correlated with more major cardiovascular adverse events and lower long-term survival in CKD patients [40,41,42], but the exact relationship between AKI and TMAO levels still lacks well-established evidence.

In addition to uremic toxins directly produced in the intestine, some drugs used in kidney patients also play roles in the gut-kidney axis and are worthy of more attention. One example is the oral iron supplement frequently used to treat anemic CKD patients. 

Oral iron boosts gut microbial protein fermentation, increases the gut-derived uremic toxins levels independent of kidney injury, and alters tight junctions in the intestines, leading to increased intestinal permeability and facilitating bacterial translocation [43]. Moreover, the oral iron supplement also prolongs gut transit time, increasing the amounts of uremic toxins, such as phenols and indoles [44,45].

#### 2.2.2. Short-Chain Fatty Acids

SCFAs are volatile fatty acids produced from indigestible food in the colon by the gut microbiota. They are fermentation products with straight and branched-chain conformation that contain fewer than six carbons and play different biological roles. In the SCFA family, propionate, acetate, and butyrate are the most known ones with crucial roles in the gut-kidney axis [46]. These SCFAs are nephroprotective compounds that might be insufficiently generated under gut dysbiosis and subsequently causes worsening AKI. Propionate mainly takes part in gluconeogenesis, whereas acetate and butyrate primarily act in lipid biosynthesis [47]. Besides energy and metabolic effects, butyrate is protective against colorectal cancer by promoting gut mobility, suppressing inflammation, and inhibiting tumor cell progression [48,49,50]. At the same time, propionate and butyrate can induce T-regulatory cell (Treg) differentiation, possibly via histone deacetylation inhibition which further mediates intestinal inflammation [51,52,53]. 

Understanding the distinctive pathways of these SCFAs’ actions sheds light on the intricate relationship between AKI and dysbiosis and provides a concrete base of the famous and novel treatment featuring SCFAs supplement. SCFAs serve as ligands of the G protein-coupled receptors (GPCRs), characterizing GPR41, GPR43, and GPR109 as the central activated receptors. SCFAs can cause a direct effect on Treg cells, neutrophils, monocytes, and mast cells through expressions of the GPCRs mentioned above [54,55,56]. These receptors are present in kidney and immune cells. They also distribute widely throughout our organs and systems. For example, adipocytes, neurons, and vascular cells display GPCRs [57,58,59]. The widespread quality of these receptors makes the SCFAs essential and potent in modulating the immune homeostasis of the whole body. 

In addition to modulating the immune system, SCFAs also seemed to participate in the hormonal system, such as by upregulating serotonin [60]. An animal study discovered that GPR43 knockout mice have less SCFA-induced glucagon-like peptide-1 (GLP-1), suggesting that SCFAs may closely regulate the hormonal system [61]. Olfactory receptor 78 is another GPCR expressed explicitly in the renal juxtaglomerular apparatus. They adjust renin secretion in response to acetate and propionate but not butyrate, stimulating blood pressure and kidney function [21,62]. 

Apart from the GPRs pathway, SCFAs, especially acetate, also serve as histone deacetylase (HDAC) inhibitors. Via HDAC inhibition, SCFAs modulate immune pathways and oxidant-antioxidant imbalance. They regulate epigenome and modify chromatin remodeling, which causes altered expression of immune-regulating genes [63]. The most well-known impact is the nuclear factor kappa-light-chain-enhancer of the activated B cells (NF-KB) signaling pathway. However, proliferator-activated receptor gamma (PPARr), IFN-γ, tumor protein p53, and the nuclear factor of activated T cells (NFAT) are also involved in the process and provide anti-inflammation protection to the kidneys [64,65]. In addition, acetate also inhibits nicotinamide adenine dinucleotide phosphate (NADPH) oxidase-2 (NOX-2)/ROS signaling through attenuating HDAC activity in T cells and resetting the tilted oxidant-antioxidant scale [66]. Another SCFA, butyrate, is also found to alter the expression of claudin-1 and the redistribution of zona occludens-1 and occludin [67]. These three molecules are essential elements in tight junction composition. Therefore, SCFAs also preserve the integrity of the intestinal wall barrier. Through GPCRs, HDAC, or other unknown routes, SCFAs play a significant role in avoiding kidney injury.

## 3. Treatment

As soon as the kidney-gut relationship has been made manifest, new treatments, especially those aiming to improve kidney injury by correcting dysbiosis, are widely introduced. The idea of these treatments probably stems from an exciting discovery in 2009 [68]. This murine IRI model experiment found that the germ-free mice had a higher natural killer T cells frequency, a higher IFN-γ expression, decreased IL-4 and IL-10 levels, and more importantly, a worse functional and structural impairment than control mice. However, conventionalizing germ mice with stools from the control group showed a more protective effect from AKI than the germ-free group [68]. The findings suggest that it might be beneficial in deterring AKI by reforming gut microbiota. The treatment strategies are listed below.

### 3.1. Prebiotics

Prebiotics was defined by the International Scientific Association of Probiotics and Prebiotics (ISAPP) in 2008 as “a selectively fermented ingredient that results in specific changes in the composition and/or activity of the GI microbiota; thus conferring benefit(s) upon host health [69]”. The beneficial effects of many prebiotics in CKD and obese settings are encouraging, including decreased uremic toxins, reduced oxidative stress, lower inflammation state, and lower mortality rates [70,71,72,73,74,75]. Table 2 summarizes the research and the results regarding these treatments.

Nonetheless, despite the promising results in CKD, the exact link between AKI and prebiotics remains unclear. An ongoing double-blind, randomized controlled trial that focuses on the effects of prebiotics and probiotics on septic AKI patients might provide a more precise answer in the future (https://clinicaltrials.gov/ct2/show/NCT 03877081, accessed on 2 January 2022).

### 3.2. Probiotics

Probiotics were defined as “the preparation of inanimate microorganisms and/or their components that confers a health benefit on the host” by the ISAPP in 2019 [76]. Unlike prebiotic treatment, several trials were conducted targeting AKI. Several probiotics, including *Lactobacillus salivarius*, *Bifidobacterium bifidum* BGN4, and microbial cocktails, show their beneficial effects for attenuating the AKI severity [77,78,79]. In contrast, the gut microbiota depletion following administration of broad-spectrum antibiotics also demonstrates protective effects against kidney injury [80]. Although the discordant results still lack a valid explanation, several hypotheses have been proposed. A potential explanation focuses on the unknown influence of antibiotics on both kidney injury and the gut. For example, is it possible that antibiotics protect AKI independent of gut microbiota depletion? Or is it possible that the broad-spectrum antibiotics preferentially deplete pro-injurious bacteria but not the protective ones [21]? In summary, probiotic treatment exhibits beneficial effects in AKI, but information regarding broad-spectrum antibiotics is partially inconclusive and necessitates further investigation. 

### 3.3. Synbiotics

A synbiotic is a supplemental treatment combining probiotics and prebiotics. The idea of getting the synergistic effect from the two therapies is rational, but the results are not satisfying. A randomized controlled trial showed that synbiotics decreased total plasma PCS concentrations but did not improve GI symptoms in non-dialyzed CKD patients [81]. Another study, the SYNERGY trial, showed a lower serum PCS level but not IS level after synbiotics treatment [82]. Although these two trials support the effect of lowering uremic toxins [81,82], the overall clinical impact of synbiotics in CKD patients is inconclusive [44]. As for AKI, the evidence is still lacking to generate a conclusion.

### 3.4. Postbiotics

The term postbiotic describes nonviable bacteria or metabolic byproducts from probiotic microorganisms, such as vitamins, SCFAs, and enzymes that can cause a positive effect on the gut microbiota and the host [83]. As listed in Table 3, many experimental studies showed promising results in improving outcomes of AKI subjects [44]. Two studies disclosed that the protective effect of postbiotics is closely associated with reduced local and systemic inflammation levels, oxidative stress, cell infiltration or activation, apoptosis, and increased autophagy. Furthermore, boosting mitochondrial biogenesis via epigenetic modification and NF-κB inhibition were proposed as the possible underlying mechanism [84,85].

### 3.5. Adsorbent

As mentioned earlier in the paper, several gut-derived uremic toxins, such as IS and PCS, cannot be effectively removed by conventional hemodialysis due to their protein-bound quality. Therefore, oral uremic toxin adsorbents are introduced as a new strategy and solution. AST-120 is one of the marketed adsorbents with great affinity to PCS and IS [44]. It shows the trend of slowing CKD progression in some phase II and phase III trials, but more concrete data remains absent [88].

Regarding AKI, administering AST-120 reduces toxin levels, weakens renal expression of mRNAs of injury-related markers, and decreases IS level in a murine AKI following myocardial infarction [87]. However, more studies are warranted before extensive application of this treatment to AKI patients.

### 3.6. Fecal Microbiota Transplantation

Fecal microbiota transplantation (FMT) is a method to restore the microbial community by introducing a healthy donor’s local gut microbes to a dysbiotic gut via either capsules or colonoscopy [83]. It has been closely linked to treating infectious diseases with dysbiotic conditions such as *Clostridioides diffcile*. Despite the protective effect of FMT shown in mice, information about FMT’s benefit on kidney disease in humans is still insufficient [80]. Two sporadic cases have been reported to discuss the tricky infectious disease in a uremic patient and a kidney transplant patient [89,90]. 

In CKD patients, we might know more in the future since a trial that aims to solve the mystery of FMT has been completed recently (https://clinicaltrials.gov/ct2/show/NCT04361097, accessed on 2 January 2022), while another trial is currently recruiting (https://clinicaltrials.gov/ct2/show/NCT04222153, accessed on 2 January 2022). Nevertheless, there are still no trials evaluating FMT’s effect on AKI.

## 4. The Unresolved Parts of Pathophysiology

Although the discovery of the Th17 pathway in the gut-kidney axis helps enlighten us a little on the adaptive immunity in AKI, there is still a great void to be filled. A previous experiment demonstrated that dendritic cells-depleted mice had worse renal tubular injury and more inflammation cells infiltration than the control group in cisplatin-induced AKI. The modulatory effect of dendritic cells on the inflammatory response may explain this unexpected effect [91]. More importantly, this may imply that dendritic cells, which act as the bridge between innate and adaptive immunity, may be the essential piece of the puzzle to complete the whole picture.

## 5. The Prospect of Future Treatment

### 5.1. Immunotherapy

Treatments aiming at the immune pathway in the kidney-gut axis are less investigated, but these immune therapies are worth anticipation. TLR-4 is a crucial player in activating the immune cells like neutrophils and macrophages in the innate immune response. The research found the required presence of TLR-4 to develop kidney injury in cisplatin-induced AKI, indicating the significance of the innate immune system in this process [92]. Regarding adaptive immunity, Th17 is regulated at different reaction levels, providing opportunities for various potential entry points for management. The most promising targets are monoclonal antibodies disrupting Th17 to produce cytokines like IL-12, IL-17A, and IL-23.

### 5.2. Repurposed Medications

In addition to the medication mentioned above, some drugs with extra-renal indications are also worth mentioning. Angiotensin II stimulates several inflammatory cytokines and adhesion molecules that participate in local inflammation [93]. Moreover, a renin-angiotensin-aldosterone system (RAAS) blockade, such as angiotensin-converting enzyme inhibitors and angiotensin II receptor blockers, can provide renal protection independent of lowering blood pressure or other hemodynamic factors [94]. Another example is allopurinol. This uric acid lowering agent inhibits xanthine oxidase activity and decreases ROS production. Since both uric acid crystals and ROS activate the NLRP3 inflammasome and set off an inflammation response, the allopurinol’s action exhibits antioxidant and anti-inflammatory effects [95]. Two recent experiments have been exhibited to slow down the inflammation and fibrosis process after allopurinol administration [96,97]. 

Given the promising antioxidant and nephroprotective effects of the RAAS blockade and allopurinol, these two repurposed medications have the potential to become optimal treatment options by blocking the vicious cycle between gut dysbiosis and AKI.

## 6. Conclusions

AKI and gut dysbiosis affect each other bidirectionally. Both macrophages and neutrophils of the innate immunity and the Th17 cell from the adaptive immunity are the critical players of AKI-induced gut dysbiosis, whereas overproduction of gut-derived uremic toxins and inadequate generation of SCFAs are the main factors deteriorating AKI. Many novel treatments are proposed to deter AKI progression by reforming the GI microbiome and breaking this vicious cycle. Existing evidence supports the benefits of probiotic treatment in AKI patients, while the beneficial effects of prebiotics, synbiotics, postbiotics, adsorbent treatment, and FMT on AKI patients are not yet strongly evident. 

In the future, intestinal microbiota and immune therapy might be two potential directions for investigation. Since gut microbiota alterations might be the hallmarks of AKI-induced dysbiosis, further experiments involving identification and transfer of gut microbiota are potential strategies for diagnosing, preventing, and even treating AKI. Regarding the immune aspect, the dendritic cells involved in the interplay of gut dysbiosis and AKI, the TLR-4 and Th17 that are crucial players in innate and adaptive immunity, respectively, the monoclonal antibodies that disrupt the cytokine production, and the renoprotective effect of the gut-derived D-serine, are suggested targets for further studies. Some repurposed medications, such as the RAAS blockade and allopurinol, are worthy of further evaluation for optimal treatment options. Furthermore, investigations are also encouraged to exclude the causal effects on gut dysbiosis of widely used drugs, such as vitamins, calcium, and erythropoietin, to diminish the unaware AKI risk in routine practice.

## Figures and Tables

**Table 1:** Studies evaluating the association between AKI and gut dysbiosis.

Studies	Subjects/Models	Results	Conclusions
Samanta et al., 2018 [14]	Wistar rats/hypoxia-induced AKI model	Under hypoxic conditions, AKI occurs, and the amount of *Escherichia coli*, *Bacteroidetes*, *Bifidobacterium*, and *Salmonella* increases in limited fecal analysis.	AKI can occur at hypobaric hypoxia and affect the gut microbial population.
Nakade et al., 2018 [15]	C57BL/6 mice/IRI model, Human	[In mice] (1) Gut microbiota protects against tubular injury in AKI mice. (2) AKI induces gut dysbiosis (increases of *Lactobacillus*, *Clostridium*, and *Ruminococcus*; and decreases *Bifidobacterium*). (3) AKI-induced gut dysbiosis alters the metabolism of D/L–amino acids ratios, causing an increased D-serine/L-serine ratio. (4) Gut-derived D-serine suppresses damage and promotes the hypoxia-mediated proliferation of tubular epithelial cells, reducing tubular injury after IRI. [In human] (5) Serum D-serine level significantly correlates with the decreased kidney function in AKI patients.	These findings show the interaction between the gut microbiota and the kidney and the renoprotective effects of gut-derived D-serine in AKI. The result also suggests D-serine as a potential new therapeutic target and biomarker for AKI.
Andrianova et al., 2020 [16]	Wistar rats/IRI model	After AKI: (1) Some gut microbiome compositions (*Rothia* and *Staphylococcus* abundance) are likely associated with AKI severity. (2) The microbiome composition is correlated with bacterial metabolites. (3) The serum levels of long-chain acylcarnitines were increased and correlated with AKI severity, whereas levels of three amino acids (tyrosine, tryptophan, and proline) had decreased.	The specific gut microbiome and metabolites might play a nephroprotective or neuropathogenic role in AKI.
Yang et al., 2020 [17]	C57BL/6 mice/IRI model	(1) AKI induces gut dysbiosis (increases of *Escherichia* and *Enterobacter*; and decreases of *Lactobacillus*, *Ruminococcaceae*, *Faecalibacterium*, and *Lachnospiraceae*). (2) Gut dysbiosis is associated with decreased levels of SCFAs, intestinal inflammation, and leaky gut. (3) Gut dysbiosis causally links to AKI severity (Mice who received post-AKI microbiota developed more severe AKI than those who received microbiota from sham-operated mice). (4) Microbiota depletion by oral antibiotics protects against kidney IRI.	(1) Hallmarks of AKI-induced dysbiosis included relative increases of *Escherichia* and *Enterobacter* and relative decreases of *Lactobacillus*, *Ruminococcaceae*, *Faecalibacterium*, and *Lachnospiraceae.* (2) Gut dysbiosis, inflammation, and leaky gut are consequences of AKI, and also essential modifiers determining post-AKI severity.

Abbreviations: AKI: acute kidney injury; IRI: ischemic/reperfusion injuries; SCFA: short-chain fatty acid.

**Table 2:** Studies evaluating the association between prebiotics and kidney function.

Studies	Subjects	Intervention	Findings
Wanchai, K. et al., 2018 [70]	Obese rats	Xylooligosaccharide	Xylooligosaccharide decreases renal oxidative stress and apoptosis
Bliss, D.Z. et al., 1996 [71]	CKD patients	Gum arabic fiber	Gum arabic fiber with a low-protein diet decreases serum urea nitrogen levels
Meijers et al., 2010 [72]	HD patients	Oligofructose-enriched inulin	Oligofructose-enriched inulin reduces uremic toxin levels
Krishnamurthy et al., 2012 [73]	CKD patients	High total fiber intake	High total fiber intake decreases the risk of inflammation and all-cause mortality
Sirich et al., 2014 [74]	HD patients	Resistant starches	Resistant starches reduce serum uremic toxin levels without intensifying dialysis treatment
Chiavaroli et al., 2015 [75]	CKD patients	Resistant starches	Resistant starches reduce serum urea and creatinine levels

Abbreviation: CKD: chronic kidney disease; HD: hemodialysis.

**Table 3:** Studies evaluating the association between AKI and postbiotics/AST-120.

Studies	Subjects	Intervention	Findings
Machado et al., 2012 [85]	Wistar rats	SCFA (Sodium butyrate)	Sodium butyrate inhibits NF-κB expression and protects against CIN
Sun et al., 2013 [86]	Sprague-Dawley rats	SCFA (Sodium butyrate)	Sodium butyrate decreases gentamicin-induced nephrotoxicity by enhancing renal antioxidant enzymes activity and the expression of prohibitin protein.
Andrade-Oliveira et al., 2015 [84]	C57BL/6 mice	SCFAs (acetate, butyrate, propionate)	Acetate diminishes inflammation in kidney epithelial and immune cells and ameliorates kidney ischemia/reperfusion injury, most likely through modulation of epigenetic processes
Fujii et al., 2016 [87]	SH rats	AST-120	Treatment with AST-120 may have protective effects on kidney injury after myocardial infarction by suppressing oxidative stress.
Al-Harbi et al., 2018 [66]	BALB/c mice	SCFA (sodium acetate)	Acetate might be beneficial during sepsis-induced AKI by restoring oxidant-antioxidant balance in T cells.

Abbreviation: AKI: acute kidney injury; CIN: contrast-induced nephropathy; NF-KB: nuclear factor kappa-light-chain-enhancer of activated B cells; SCFA: short-chain fatty acid.

## Data Availability

Not applicable.
